# Baseline assessment of prevalence and geographical distribution of HPV types in Chile using self-collected vaginal samples

**DOI:** 10.1186/1471-2458-8-78

**Published:** 2008-02-28

**Authors:** Catterina Ferreccio, Alejandro Corvalán, Paula Margozzini, Paola Viviani, Claudia González, Ximena Aguilera, Patti E Gravitt

**Affiliations:** 1 P. Universidad Católica de Chile, Escuela de Medicina, Santiago Chile; 2 Departamento de Epidemiología, Ministerio de Salud, Santiago Chile; 3 The Johns Hopkins University, Bloomberg School of Public Health. Baltimore, USA

## Abstract

**Background:**

Chile has broad variations in weather, economics and population from the far desert north (Region 1) to the cold, icy south (Region 12). A home-based self-collected vaginal sampling was nested in the 2003 Chilean population-based health survey in order to explore the possibility of a type-specific geographical variation for human papillomavirus

**Methods:**

The population was a national probability sample of people 17 years of age and over. Consenting women provided self-collected cervicovaginal swabs in universal collection media (UCM). DNA was extracted and typed to 37 HPV genotypes using PGMY consensus PCR and line blot assay. Weighted prevalence rates and adjusted OR were calculated.

**Results:**

Of the 1,883 women participating in the health survey, 1,219 (64.7%) provided a cervicovaginal sample and in 1,110 (56.2% of participants and 66.5% of those eligible) the samples were adequate for analysis. Refusal rate was 16.9%. HPV prevalence was 29.2% (15.1% high-risk HPV and 14.1% low-risk HPV). Predominant high-risk types were HPV 16, 52, 51, 56 and 58. Predominant low-risk HPVs were HPV 84, CP6108, 62, 53 and 61. High-risk and low-risk HPV rates were inversely correlated between the regions. High-risk HPV prevalence was highest among the youngest women, whereas low-risk HPV increased slightly with age.

**Conclusion:**

Self-obtained vaginal sampling is adequate for monitoring HPV in the community, for identifying high-risk areas, and for surveying the long term impact of interventions.

## Background

The availability of highly effective human papillomavirus (HPV) vaccines has led to a discussion about their incorporation in public health vaccine programs [1,2]. A baseline characterization of HPV types in cervical cancers and also in the general population is required before deciding whether to introduce the vaccine. With this information on the population genotype prevalence, together with regional specific genotype distribution in cervical lesions and cervical cancer, the potential impact of the vaccine can be estimated. In a previous study of HPV prevalence in cervical specimens of a population-based sample of women, we explored the individual risk factors for low-risk and high-risk HPV infections, confirming that in Chile the main risk factor was the number of sexual partners for both high and low risk HPV [3]. This study aims to describe the geographical distribution of the HPV types in the national community. Epidemiologic studies of the prevalence of HPV in the community have typically used cervical samples obtained by a gynaecological examination [4]. Most women find this exam to be uncomfortable [5]. Additionally, in countries where Papanicolaou (PAP) screening is not widely accepted, studies based on gynaecological sampling may not represent the general population. For community surveillance, public health officers will need non-invasive methods that are acceptable to a representative sample of women. One option is the self-obtained vaginal sample. Various well-designed trials have demonstrated that HPV detection using self-obtained vaginal sample is a reasonable surrogate for the identification of HPV types that are infecting the cervix, with moderate to excellent agreement with the detection of HPV from cervical samples obtained by clinicians [6-10]. Agreement was better for the high-risk HPVs [6,8,11,12]. Studies comparing acceptability of methods demonstrated that most women preferred self-vaginal sampling [5,8,13-19]. We assessed the prevalence and distribution of HPV types throughout Chile using self-obtained vaginal sampling in the community. The National Health Survey, ENS 2003, the first prevalence study of chronic diseases, provided a population-based national sample of non-institutionalized people aged 18 and above [20]. This is one of the first studies with self-obtained vaginal sampling in a health survey for chronic conditions in the adult population. The aims were to assess the magnitude of the problem and to explore the association of HPV prevalence with some regional characteristics.

**Figure 1:**
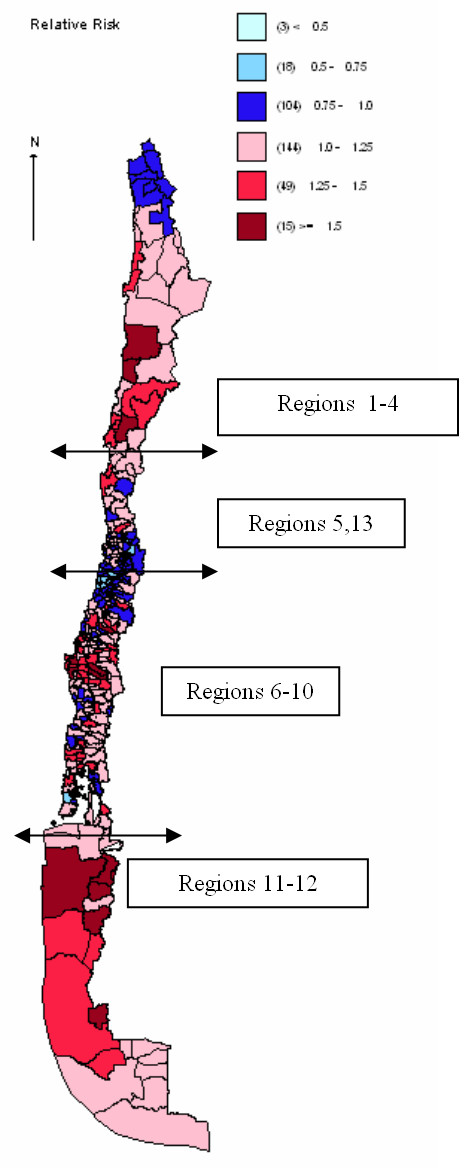
Relative Risk of cervical cancer mortality by county of residence. Chile average 1985–2002^(1) ^1: Prepared by the authors based on Ministry of Health mortality and population data.

Chile is a long (6,435 km length) and narrow (200 km wide) country (Figure 1) with a climate that goes from the driest desert in the world, with a mean temperature of 18.1°C and 0 mm of rain/year in the extreme north (Region 1) to a cold, windy and rainy (3,500 mm yearly) climate in the far south (Region 12). The population in the northern regions resides mostly in urban areas, with a racial admixture of Hispanic and Andean Indians. Its main economic activity is copper mining. Most of the Chilean population lives in the central regions. The metropolitan area (Region 13) is located between Regions 5 and 6; the climate is temperate with an average of 15°C and an average annual rainfall of 150–200 mm. Its main economic activity is industry and wine production. The population is an admixture of Spanish with native Mapuche Indians. The southern regions are mountainous, with many lakes and rivers, and large rural population. Average rainfall is 1,500–2,000 mm/year, with a temperature range of 2°C-23°C. This area hosts the largest ethnic Mapuche population and is the poorest in the country. Its main economic activity is agriculture and livestock production. The far southern regions have a cold climate and their principal activity is fisheries; its native population is the lowest in the country.

## Methods

### Population and sample

The study population included all women participating in the ENS 2003. The sample for the ENS 2003 was a national stratified multistage probability sample of non-institutionalized people aged 18 years and above, representing the Chilean population and comparing regions, rural and urban, four age groups, and three socio-economic strata. The sampling error was set at less than 2%. The ENS 2003 is the first household based health survey with biological sampling (blood, urine) body measurements (height, weight, waist circumference), clinical examinations (visual and auditive acuity, dental health) and comprehensive health questionnaires administered by a public health certified nurse and a trained interviewer. Only one subject, selected by the Kish method, participated per household [21]. The survey was conducted from June to December 2003. The ethics committees of the Catholic University and the Ministry of Health reviewed and approved the study. All women signed a separate informed consent for the self-obtained vaginal sample.

### Self-obtained vaginal sampling

After receiving information about the exam and providing written informed consent, eligible women who were not menstruating, not pregnant, had not had sexual relations the previous day, with no prior hysterectomy, and had initiated sexual activity were taught, using pictorial pamphlets, how to obtain a vaginal sample. The nurse provided a Digene sachet containing a conical brush and a tube with the transport media (Universal Collection Media, UCM) [22]. Women were instructed to introduce the brush deep into their vagina and turn it smoothly once towards the left and then towards the right. They then were instructed to place the brush in the tube, cover it with a lid and give it to the nurse. Tubes were transported and stored at 4°C until processing. Most samples were processed 35 days after the exam was taken, with a range of 10 days to four months, with no difference in positivity rate with regard to the time elapsed before processing. A PAP screening was recommended for women with an HPV positive result.

### Laboratory analysis

In the Catholic University Pathology Laboratory, DNA from the self-obtained vaginal sample was extracted with the DNeasy tissue kit (Qiagen, Valencia, CA USA) following the manufacturer's instructions. All extracted DNA samples were sent to Johns Hopkins University where they were typed using PGMY consensus PCR and prototype line blot assay (Roche Molecular Systems) to identify 37 HPV genotypes [23-25]. DNA extraction, PCR set-up and amplification/detection were conducted in separate laboratories using dedicated instruments to minimize contamination. Specimens were opened only after brief centrifugation using clean Kim-wipe to avoid specimen-to-specimen carryover. Each 96 well PCR plate included eight DNA negative controls. HPV 16 and 18 positive plasmid controls in a background of 50 ng/ml human placental DNA at high (approx 5000 copies/PCR) and low (approx 25 copies/PCR) concentration were also included in each PCR assay. Two negative controls per detection tray were included to ensure no spill over during hybridization.

### Collection of secondary data from Chilean Regions

To explain the variation in the prevalence of HPV among the 13 Chilean Regions, we collected available regional population data for women's characteristics (fertility rate, educational level, smoking rate), socio-demographic characteristics (life expectancy, percent Amerindians, percent single, poverty index, age distribution), access to health care (Papanicolaou coverage), HIV prevalence rate (indicator of sexually transmitted diseases) and cervical cancer mortality. We obtained mortality data and health information from the Ministry of Health and population data from the National Institute of Statistics.

### Statistical analysis

Prevalence rates were calculated using sampling weights based on the two stage sampling design and adjusted for post stratification population totals using the Chilean 2003 population statistics. Considering the complex sample design, the standard error and 95 percent confidence intervals were calculated with the Taylor linear approximation method using SPSS version 14 [26]. Adjusted OR for high-risk and low-risk infections were calculated from a Multivariate Logistic Regression Model for Complex Samples. Age adjusted high risk and low risk HPV prevalence, were calculated with a general additive model using R software. For the exploratory regional-level analyses of HPV and some regional characteristics partial correlations were used adjusted by age and multivariate linear models using SPSS 14.

## Results

### Survey response rates

A total of 1,883 women participated in the ENS2003; 12% were not eligible – reasons for ineligibility included women currently menstruating, having had sexual intercourse less than 24 hours previously (9.1%), or prior hysterectomy (2.9%); 8.1% could not be included because the interviewers did not have swabs when conducting the survey; 16.9% refused to take the self-sample leaving a total of 1,219 (64.7%) women who provided a self exam. 109 (8.9%) of the samples were insufficient for HPV analysis as indicated by the absence of the human beta-globin control amplification (1.6%) or were untested because the tube was broken or empty. Therefore, 1,110 women (56.2% of the participants and 66.5% of the eligible women) provided an analyzable vaginal sample. Women refusing to collect the sample were similar to those who accepted the exam with regard to region of residence and urban-rural zone, but higher refusals were observed among the younger (< 25 years old), the older (> 60 years old), the lower educational group, the single, and the widowed.

### Population characteristics

The mean age of the survey participants was 41.9 years (range 16–97) and the majority lived in urban areas (88.1%) (Table 1). The mean number of years of education was 9.4 (range 0–22); 26% had less than 4 years of education. The majority of participants (62.5%) were unemployed (i.e., not working or studying) and were married or cohabiting (66%). The survey population was reasonably well-screened, with 85% of women reported having a previous Papanicolaou smear (62% in less than 3 years), and 2.6% reporting having had a previous abnormal Papanicolaou smear. The regional distribution of participants represents the baseline population.

**Table 1:** Characteristics of the 1 110 women studied and weighted percents.

**Characteristics (n)**	(n) weighted percent *
**Age (years)**	
18–24	(102) 13.1
25–29	(73) 10.2
30–34	(104) 13.4
35–39	(90) 11.6
40–44	(114) 11.6
45–49	(129) 10.7
50–54	(109) 8.4
55–59	(94) 6.2
60–64	(83) 5.8
> = 65	(212) 9.0
**Education**	
High	(128) 16.6
Medium	(556) 57.2
Low	(426) 26.2
**Marital status**	
Married/Cohabiting	(674) 65.8
Widow	(121) 5.3
Separated	(85) 5.7
Single	(228) 23.1
**Smoker**	
No	(735) 60.1
Yes	(367) 39.9
**Pap ever**	
Yes	(939) 84.6
No	(167) 15.4
**Residence**	
Urban	(947) 88.1
Rural	(163) 11.9
**Region**	
North	12.0% (10.5–13.7)
Central	54.0% (50.3–57.6)
South	32.9% (29.7–36.3)
Extreme South	1.1% (0.8–1.4)

*::using sampling weights and adjusted for post stratification population totals.

### Acceptability of self-sampling

Most women [89.6%, (95% CI 86.8–91.9)] reported no discomfort with the self-administered swabs and the majority [79.4% (95%CI 75.9–82.6)] indicated self sampling caused less discomfort than the Papanicolaou smear (manuscript in preparation).

### HPV prevalence: Regional variation by risk group

Overall, we observed 29.2% point prevalence for any HPV infection in a population-based survey of adult Chilean women. Of the infected women, 146 (15.3%) had at least one high risk HPV genotype detected and 83 (8.4%) had exclusively high-risk HPV types. Only low-risk HPV types were detected in the remaining infected women (162, or 13.9% of the survey population). A total of 488 infections were identified (some women may be infected with more than one HPV type); of those 202 (41.4%) were caused by high-risk HPVs and 286 (58.6%) by low-risk HPVs. HPV 16 was the predominant type of high-risk HPV, followed by HPV 52, 51, 56 and 58. Among the low-risk HPVs the most frequently detected types were HPV 84, CP6108, 62, 53 and 61 (Table 2).

**Table 2:** HPV infections by HPV types in vaginal samples of 1,110 women from the National Health Survey 2003.

**HPV type**	**Single**	**Multiple**	**Total (crude %)**	% adjusted*	**95% CI adjusted**
HPV -			802 (72.3)	70.8	(66.3–74.9)
HPV +	208	100	308 (27.7)	29.2	(25.1–33.7)
HR HPV+	70	76	146 (13.2)	15.3	(11.9–19.3)
HR HPV only	70	13	83 (7.5)	8.4	(5.9–11.8)
LR HPV+ only	138	24	162 (14.6)	13.9	(11.2–17.2)
HR infections					
16	19	10	29 (2.6)	3.2	(1.6–6.3)
18	1	5	6 (0.5)	0.3	(0.1–0.8)
31	5	7	12 (1.1)	1.2	(0.6–2.5)
33	1	5	6 (0.5)	0.4	(0.2–1.2)
35	4	6	10 (0.9)	1.1	(0.5–2.2)
39	4	7	11 (1.0)	1.3	(0.5–3.2)
45	5	7	12 (1.1)	0.9	(0.4–1.8)
51	6	11	17 (1.5)	2.1	(1.0–4.3)
52	5	19	24 (2.2)	2.6	(1.3–4.9)
56	4	11	15 (1.4)	2.0	(0.8–4.9)
58	2	17	19 (1.7)	1.7	(1.0–3.0)
59	7	4	11 (1.0)	1.6	(0.7–3.3)
66	3	9	12 (1.1)	1.6	(0.6–4.0)
68	3	5	8 (0.7)	0.5	(0.2–1.2)
73	1	9	10 (0.9)	1.0	(0.5–2.3)
Subtotal	70	132	202		
LR infections					
6	5	4	9 (0.8)	1.2	(0.5–2.6)
11	1	0	1 (0.1)	0.1	(0.0–1.0)
26	1	1	2 (0.2)	0.1	(0.0–0.4)
40	1	2	3 (0.3)	0.2	(0.0–0.8)
42	2	10	12 (1.1)	1.2	(0.6–2.4)
53	8	16	24 (2.2)	2.6	(1.2–5.6)
54	2	6	8 (0.7)	0.3	(0.1–0.7)
55	4	3	7 (0.6)	0.6	(0.2–1.7)
61	20	15	35 (3.2)	2.3	(1.4–3.8)
62	10	16	26 (2.3)	3.1	(1.8–5.3)
64	3	0	3 (0.3)	0.1	(0.0–0.3)
67	4	5	9 (0.8)	1.4	(0.5–4.0)
70	9	12	21 (1.9)	1.8	(1.1–3.1)
71	6	13	19 (1.7)	1.3	(0.7–2.7)
72	10	1	11 (1.0)	1.0	(0.4–2.2)
81	9	9	18 (1.6)	2.0	(1.0–4.0)
83	8	4	12 (1.1)	0.8	(0.3–1.7)
84	21	14	35 (3.2)	3.7	(2.3–6.1)
CP6108	12	14	26 (2.3)	3.2	(1.8–5.5)
IS39	2	3	5 (0.5)	0.8	(0.2–2.3)
Subtotal	138	148	286		

Total	208	280	488		

HR: high-risk, LR: low-risk; *::using sampling weights and adjusted for post stratification population totals

**Figure 2:**
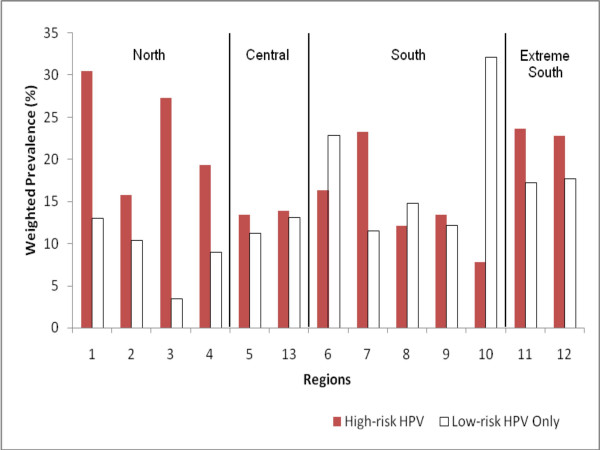
Regional distribution high-risk and low-risk HPV in the population of Chile's 13 Regions.

Total HPV infection (high-risk plus low-risk HPVs) varied from 43.5% (95% IC, 23.5–65.9) in Region I (the northernmost region) to 25% (96% CI, 15.6–36.6) in Region V (Figure 2). Prevalence of high-risk (either single or multiple) and low-risk HPVs (single or multiple low-risk only) rates were inversely correlated between the regions (Pearson correlation -0.62 p= 0.023). Although not statistically significant the age distribution varied between the regions with the lowest mean age in Region I and the highest in Region XII (mean age in years 38.6 and 45.0, respectively). When weighted and age-adjusted, this inverse association between high and low-risk HPVs decreased and lost its statistical significance (age adjusted partial correlation -0.36 p = 0.25). Based on the relative contribution of a high-risk HPV to the total burden of HPV, four distinct macroregions were identified: the North (adjacent to Peru and Bolivia), Central, South, and Extreme South (Patagonia). The OR of having high-risk HPVs was **2.8 (95% CI 1.4–5.7)**, 0.9 (95% CI 0.6–1.5), 0.6 (**95% CI 0.3–0.98**) and 1.09 (95% CI 0.4–3.0), for the North, Central, South and Extreme South areas respectively. The mean ages of the population were: 39.8, 42.7, 41.1 and 40.0 for North, Central, South and Extreme South regions, respectively. Neither high-risk HPV nor low-risk HPV was associated with cervical cancer mortality (Pearson correlation 0.2 and 0.1 respectively; test for trend, *p *= 0.29 and 0.37, respectively). In 2004, according to the ministry of health statistics, the Papanicolaou coverage for women 35–64 years old varied from 54.7% in the Metropolitan South-eastern Health Service to 87.3% in the Metropolitan Central Health Service. Nevertheless, Papanicolaou coverage was not significantly associated with either high-risk (Pearson correlation 0.15 *p *= 0.6) or low-risk HPV (Pearson correlation -0.35 *p *= 0.23).

The prevalence of current smokers, birth-rate and male-female ratio (number of men divided by number of women in the baseline population) correlated significantly with the prevalence of high-risk HPV (Pearson correlation and p values 0.63, 0.01; 0.47, 0.05 and 0.47, 0.05, respectively). Prevalence of current smokers, prevalence rate of HIV and proportion of singles, were inversely correlated with low-risk HPV (Pearson correlation and p value -0.45, 0.059; -0.50, 0.04, respectively and 0.43, 0.069).

### Individual risk factors for HPV infection

**Figure 3:**
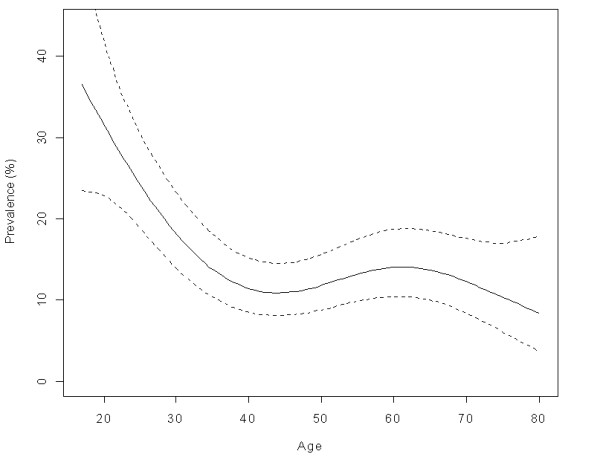
Age prevalence of high-risk HPV in vaginal samples from the population. National Health Survey, Chile 2003.

**Figure 4:**
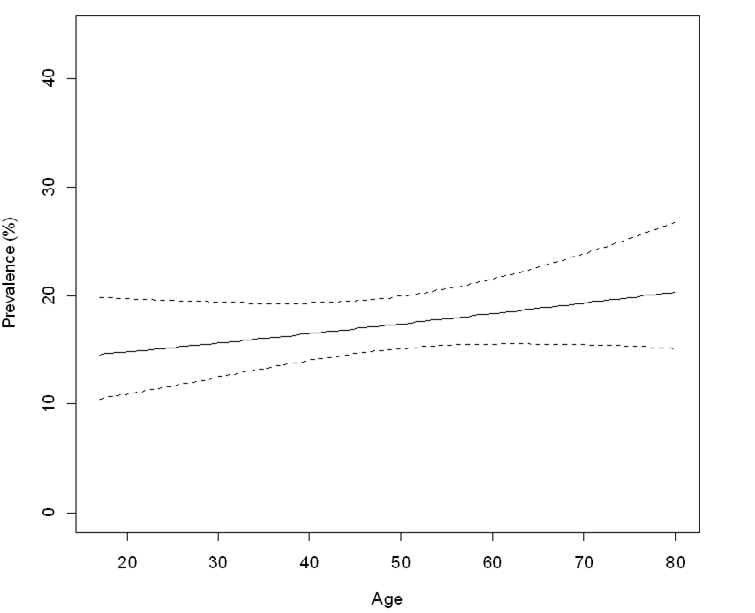
Age prevalence of low-risk HPV in vaginal samples from the population. National Health Survey, Chile 2003.

High-risk HPV prevalence was highest in the younger age group (under 25 years old) and declined significantly with age (Figure 3). Low-risk HPV had a positive but not statistically significant increase with age (Figure 4). Single women had a higher prevalence of high-risk HPV infection (OR = 3.7 95% confidence interval (CI) 2.1–6.6) while women who were older (OR = 0.29, 95% CI; 0.1–0.8) and living in a rural area (OR = 0.25 95% CI 0.1–0.7) were significantly less likely to have prevalent high-risk HPV (Table 3). Ever having had a Papanicolaou was unrelated with either high-risk or low-risk HPV. We found no association between either high-risk or low-risk HPV and years of education. After multivariate adjustment, the only risk factors for low-risk HPV were being separated (OR = 2.1 95% CI 1.2–3.7) and residing in the southern regions (OR = 2.3 95% CI 1.4–3.7) (Table 3).

**Table 3:** Risk factors for HPV infection among the 1 110 women, National Health Survey, Chile 2003.

**Characteristics (n)**	**HR HPV**	**Crude OR**	**Adjusted OR**	**LR HPV only**	**Crude OR**	**Adjusted OR**
	**(95% IC)**	**(95% IC)**	**(95% IC)**	**(95% IC)**	**(95% IC)**	**(IC 95%)**
	**n = 146**			**n = 162**		
**Age (years)**						
18–29	26.8	1.0	1.0	13.7	1.0	1.0
	(18.5–37.1)			(8.2–22.0)		
30–49	13.9	**0.5**	0.7	13.0	1.3	0.8
	(9.1–20.7)	**(0.3–0.9)**	(0.4–1.4)	(9.2–18.1)	(0.7–2.4)	(0.3–2.0)
50–64	8.5	0.8	**0.4**	15.6	1.3	0.9
	(5.7–12.5)	(0.4–1.4)	**(0.2–0.99)**	(10.4–22.6)	(0.7–2.6)	(0.3–2.3)
> = 65	8.0	**0.4**	**0.3**	16.1	1.8	0.9
	(4.6–13.5)	**(0.2–0.99)**	**(0.1–0.8)**	(10.7–23.6)	(0.8–3.8)	(0.3–2.4)
**Education**						
High	17.0	1.0	1.0	16.2	1.0	1.0
	(9.2 – 29.3)			(8.5–28.5)		
Medium	16.7	0.8	1.2	12.6	0.7	0.7
	(12.0 – 22.8)	(0.4–1.3)	(0.5–2.9)	(9.5–16.6)	(0.4–1.2)	(0.3–1.6)
Low	11.0	0.9	1.4	15.4	0.6	0.8
	(7.6 – 15.8)	(0.5–1.7)	(0.6–3.8)	(11.0–21.2)	(0.3–1.1)	(0.4–2.0)
**Marital st**	10.0	1.0	1.0	12.9	1.0	1.0
Married/Cohabiting	(6.7–14.7)			(9.7–17.1)		
Widow	12.9	**2.0**	2.3	21.4	**1.8**	1.9
	(6.7–23.1)	**(1.02–3.9)**	(0.9–5.4)	(13.5–32.2)	**(1.1–3.3)**	(0.9–3.7)
Separated	6.8	1.1	0.7	26.5	**2.1**	**2.5**
	(3.0–14.4)	(0.5–2.3)	(0.2–2.0)	(14.8–42.6)	**(1.2–3.7)**	**(1.1–5.7)**
Single	33.2	**2.7**	**3.7**	12.3	1.3	0.9
	(24.8 – 42.8)	**(1.7–4.2)**	**(2.1–6.6)**	(7.6–19.2)	(0.83–2.2)	(0.5–1.7)
**Smoker**						
No	11.8	1.0	1.0	13.9	1.0	1.0
	(8.3 – 16.6)			(10.6–18.1)		
Yes	20.6	1.3	1.46	14.1	1.18	1.1
	(14.5 – 28.4)	(0.9–1.9)	(0.8–2.8)	(10.0–19.6)	(0.8–1.7)	(0.6–1.9)
**Pap ever**						
Yes	14.0	1.0	1	13.9	1.0	1.0
	(10.4–18.6)			(10.9–17.5)		
No	21.9	0.99	0.8	14.0	1.13	0.9
	(13.6–33.3)	(0.6–1.7)	(0.4–1.7)	(8.4–22.4)	(0.7–1.9)	(0.4–1.9)
**Residence**						
Urban	16.7	1	1.0	13.6	1.0	1.0
	(12.9 – 21.2)			(10.7–17.1)		
Rural	5.0	**0.4**	**0.2**	16.8	1.3	1.08
	(2.1 – 11.2)	**(0.2–0.8)**	**(0.1–0.7)**	(10.2–26.5)	(0.8–2.1)	(0.6–2.1)
**Region**						
North	22.7	1.0	1.0	8.5	1.0	1.0
	(16.6–30.3)			(4.5–15.6)		
Central	13.9	**0.5**	0.6	12.8	1.7	1.5
	(9.0–20.8)	**(0.3–0.9)**	(0.3–1.1)	(8.9–18.1)	(0.9–3.0)	(0.7–3.5)
South	14.5	**0.6**	0.7	18.0	2.3	**2.4**
	(9.9–20.6)	**(0.4–0.88)**	(0.4–1.3)	(14.0–22.9)	**(1.4–3.7)**	**(1.1–5.0)**
Extreme South	24.7	0.83	1.5	15.9	1.9	1.9
	(11.8–44.7)	(0.4–1.8)	(0.5–4.6)	(6.9–32.4)	(0.8–4.6)	(0.6–5.9)

## Discussion

Surveillance of adult health status and its risk factors has become an essential tool for public health planning and evaluation. To date this surveillance has focused on cardiovascular diseases and its risk factors and, therefore, this is one of the first surveys to incorporate HPV testing as one of the priority problems for adult health surveillance. The overall prevalence of any HPV was strikingly similar to a recent US national survey using similar methods; however we did note an interesting contrast between high-risk and low-risk HPV prevalence and regional variability that was not previously reported [27].

In the multivariate analysis, residing in the north of Chile was a risk factor for high-risk HPV infection. The relative contribution of high risk HPV to the total burden of HPV infection was almost 3 times higher in the north than the national average while in the south it was almost half the national average [OR 2.8 (95% CI, 1.4–5.7) and 0.6 (95% CI, 0.3–0.98), respectively]. The relative excess of high-risk types in the north appears to be associated with a younger age and with variations in factors associated with sexual behaviour. While we could not explore sexual behaviour directly, secondary data shows that northern regions have higher rates of smoking, higher birth rates and higher rates of HIV while they are less rural and have less poverty than the southern regions (data not presented). On the other hand, the excess of low- risk HPV in the south appears to be associated with older age, poverty and rurality. Other authors have also described a diverse epidemiological profile for low and high risk HPVs [4,28]. To explain the differences in the relative distribution of high and low risk HPV, some authors have suggested an antagonism between high-risk and low-risk HPVs. In a serologic case-control study, Luostarinen, et al. [29] describe a statistically significant null association with cervical cancer risk among women who are seropositive to both HPV 6/11 (OR = 1.0) and HPV 16, relative to HPV 6/11 alone (OR = 2.2) or HPV 16 alone (OR = 5.5). Similarly, Wheeler, et al. [30] observed that women who had no HPV had a five times higher probability of acquiring HPV 16 in the following two years than women who carried low-risk HPVs (authors' calculation from Table 2 of Wheeler 2006). These reports, coupled with our ecologic observational data of an inverse correlation between low and high risk HPV infections, not entirely controlled by age adjustment, point to possible immunological interactions between the cervicovaginal HPV flora that require further investigation.

The age-specific prevalence of HPV were the same in the current survey and in our previous study, with high-risk HPV prevalence peaking below 25 years old while low-risk HPV increased slowly after age 50 [3]. The increase in the prevalence of low-risk HPV in older ages has been reported in some studies in Latin America and Spain [31-33] and confirmed in a recent meta-analysis [34], whereas others have found a flat age curve for low-risk HPV [25,35,36]. Some authors have attributed the excess of low-risk HPV in older women to the elimination of high-risk HPV by the screening programs [35]. It is intriguing that while low-risk HPV incidence at younger ages is half as frequent and in some reports is undetectable twice as fast as high-risk HPV [37-39], it increases in frequency at older ages both in screened and unscreened women [40]. A recent study by Wheeler found the same 2 year clearance rate for low-risk (36%), high-risk except 16 (41%) and HPV 16 (34%) (calculated from Table 3 in Wheeler 2006). The possibility that low-risk HPV in adult populations results from frequent reactivation or re-infection should be considered in view of these data.

**Figure 5:**
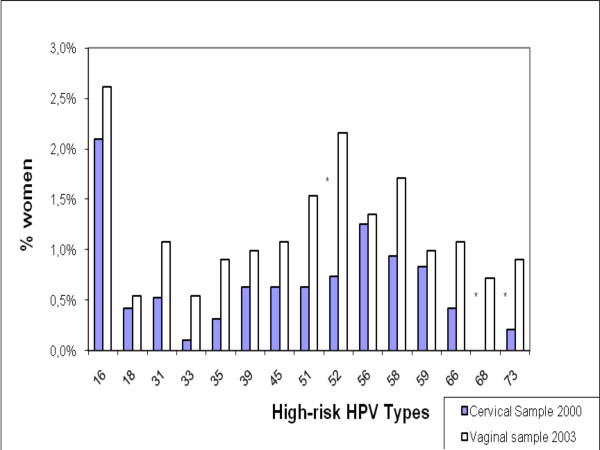
Types of High-risk HPV isolated from cervical samples in 2000 in Santiago and from vaginal self-collected samples in 2003 all Chile. **Note**: the asterisks denote a significant Chi square test with a p value <0.05 for the difference in the proportion between cervical and vaginal samples.

**Figure 6:**
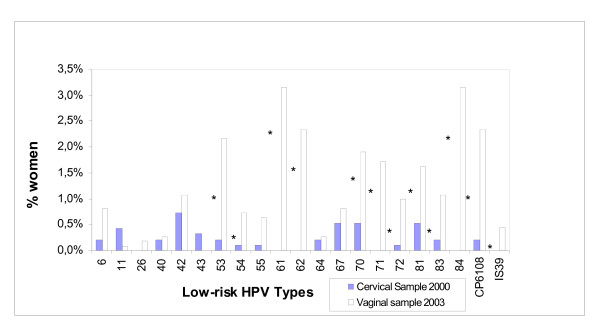
Types of low-risk HPV isolated from cervical samples in 2000 in Santiago and from vaginal self-collected samples in 2003 all Chile. **Note**: the asterisks denote a significant Chi square test with a p value <0.05 for the difference in the proportion between cervical and vaginal samples.

When comparing the HPV types found in these vaginal samples with the published data on HPV types isolated from cervical samples in Chile's general population [3] we found a remarkable coincidence in the distribution of high-risk HPV types (Fig 5) in vaginal and cervical swab samples. However, we found no similar correlation in the most frequent low-risk types (Figure 6). HPV DNA positivity was assessed in the cervical sample using a general primer-mediated GP5+/6+ polymerase chain reaction (PCR) and an Enzyme Immune Assay (EIA), to detect 36 HPV types (Ferreccio 2004), while in the current study, as already described, DNA was extracted and typed using PGMY consensus PCR and line blot assay to 37 HPV genotypes. However, these methods have been shown to have similar test characteristics, with difference in the detection of only 3 types: 35, 53 and 61 [41]. These results suggest that while self-collected vaginal swabs may result in higher low-risk HPV prevalence estimates compared to cervical swab samples, the concordance for high-risk, clinically relevant HPV is high [9,12,42].

Self-sampling was highly acceptable for Chilean women, as has been repeatedly demonstrated in different cultural settings in Canada [18], USA [16], Germany [13], Mexico [5], Brazil [19], Africa [8] and China [43]. Collectively, these data suggest that vaginal self-collected swabs could be effectively used for primary cervical cancer screening via HPV DNA testing. This application was particularly useful for those women who had never been screened, 70% of whom agreed to self-sample in our survey.

There are several caveats in the interpretations and generalizations based on this population-based survey of HPV type-specific prevalence. The relatively low response rate may bias the results. Nevertheless, since the non-participants are weighted, part of the bias may have been removed. We estimated the bias by correcting the estimates based on propensity scores, but the estimated prevalence changed minimally (Vives A. manuscript in preparation), therefore we believe that these biases do not significantly change our inferences.

The most important limitation in estimating the real HPV burden is the fact that we are measuring HPV at a single point in time, with no information about the historical exposure of these women to HPV. Winer and colleagues [44] estimated a cumulative HPV incidence of 32% after 2 years of follow-up among young college women. HPV seroprevalence (cumulative lifetime exposure estimate) have been generated from a population survey [45,46], with an average 15% – 25% cumulative exposure to HPV 16 alone. Compared to the 3.2% HPV 16 DNA observed in this population, clearly cervicovaginal HPV DNA point prevalence is not a valid marker of ever having been exposed to the virus.

## Conclusion

Self-obtained vaginal sampling is an adequate method for monitoring the burden of HPV in the community. Coupling this sample collection with routine census procedures allows high-risk areas to be identified in order to prioritize interventions and to carry out routine surveillance to assess the impact of the interventions (e.g., HPV prophylactic vaccination) over time.

## Competing interests

The author(s) declare that they have no competing interests.

## Authors' contributions

CF conceived the study, and participated in its design and coordination and drafted the manuscript, AC carried out the extraction of DNA and coordinated the lab work, PM participated in the design of the study and coordination of field work, PV performed the statistical analysis. CG participated in study design and coordination, XA participated in study design and coordination, PG carried out the molecular studies and helped to draft the manuscript. All authors read and approved the final manuscript.

## Pre-publication history

The pre-publication history for this paper can be accessed here:


